# Estimation of meal portions in bulimia nervosa compared to anorexia nervosa and healthy controls

**DOI:** 10.1007/s40519-022-01410-w

**Published:** 2022-05-19

**Authors:** Patrick Pasi, Mayron Piccolo, Lisa-Katrin Kaufmann, Chantal Martin-Soelch, Christoph Müller-Pfeiffer, Gabriella Milos

**Affiliations:** 1 Department of Consultation-Liaison Psychiatry and Psychosomatic Medicine, University Hospital Zurich, University of Zurich, Culmannstrasse 8, 8091 Zurich, Switzerland; 2 Department of Psychology, Harvard University, Cambridge, USA; 3 IReach Lab, Unit of Clinical and Health Psychology, Department of Psychology, University of Fribourg, Fribourg, Switzerland; 4 Division of Neuropsychology, Department of Psychology, University of Zurich, Zurich, Switzerland

**Keywords:** Anorexia nervosa, Bulimia nervosa, Eating disorders, Portion size, Meal portions, Estimation

## Abstract

**Purpose:**

Bulimia nervosa (BN) and anorexia nervosa (AN) are potentially life-threatening eating disorders (ED) that primarily affect young people, mostly women. The central common pathology is linked to the relationship with food and with abnormalities in food intake. A previous study indicated that individuals with AN tend to overestimate food portion sizes compared to healthy controls (HC), but no study has investigated these patterns in BN, which was the objective of this study.

**Methods:**

Women with BN (27), AN (28), and HC (27) were asked to rate different meal portion sizes in two conditions: as if they were supposed to eat them (intent-to-eat condition) or in general (general condition). BN results were compared to HC and AN using mixed model analyses.

**Results:**

BN showed larger estimations compared to HC, while smaller estimations compared to AN. These differences were found mostly for intermediate portion sizes. No difference for conditions (intent-to-eat; general) was found between groups.

**Conclusion:**

When estimating food portion sizes, individuals with BN seem to fall intermediately between HC and AN. ED symptoms in BN were most strongly associated with higher portion estimation. This might therefore reflect one aspect of the cognitive distortions typically seen also in AN. A therapeutic option could include the frequent visual exposure to increasing portions of food, what may serve to recalibrate visual perceptions of what a “normal-sized” portion of food looks like.

**Level of evidence:**

Level II: Evidence obtained from well-designed controlled trials without randomization.

**Supplementary Information:**

The online version contains supplementary material available at 10.1007/s40519-022-01410-w.

## Introduction

Bulimia nervosa (BN) and anorexia nervosa (AN) are potentially life-threatening eating disorders (ED) that primarily affect young people, mostly women (American Psychiatric Association, 2013). Individuals with BN and AN can show different comorbidities and problems, but the central common pathology is linked to the relationship with food and abnormalities in food intake [1].

Individuals with eating disorders are often preoccupied with calorie counting as well as with food portion sizes [2], and the selection of adequate portions sizes (i.e., selection of food quantities that allows to regulate weight in a healthy direction) play a key role in the nutritional treatment of all ED [3]. In AN, the orientation to adequate portion size is of central importance not only to reach a normal weight, but also to maintain it [3]. In the treatment of BN, keeping mealtime with appropriate food content and food size is from main significance to overcome the illness [4].

A previous study showed that the overestimation of small meal portions by individuals with AN was significantly greater in the intent-to-eat condition. Disturbed perceptions in AN seemed not only to include interoceptive awareness (body weight and shape, hunger, and satiety) but also external disorder-related objects such as meal portion size [2].

Although very important for understanding the etiology of BN, no study has evaluated food portion estimation in the disorder so far, and a recent review article underlined the lack of information on this subject [3]. It is important to assess portion estimation in BN to find out if individuals with BN share food-related cognitive anomalies with individuals with AN, according to the transdiagnostic theory of ED [5], since, following weight-recovery treatment, individuals with AN may switch to a diagnosis of BN [6]. Studies that deepened this phenomenon, known as diagnostic crossover, reported that more than one third of individuals with AN developed BN during course of illness [7–9]. Identifying markers present in the etiology of both disorders may contribute to better explanations of diagnostic crossover and help to prevent it.

Since treatment options for BN include appropriate recording of portion sizes [4], assessing which differences exist between individuals without an eating disorder and individuals with BN may be crucial for treatment outcomes. Furthermore, identifying whether similarities exist between different types of eating disorders, such as BN and AN, may increase understanding of the etiology of such disorders and improve treatment outcomes. In the present study we first compared individuals with BN and healthy controls (HC) and then BN and individuals with AN. We hypothesized that there would be a general difference in portion estimation between BN and HC, and in a smaller extent between BN and AN. Secondly, we expected this difference to be increased for BN in comparison to HC but similar to AN in the intent-to-eat condition.

## Methods and materials

*Participants *(Table 1). The present sample included 27 women with a diagnosis of current BN, 28 women with a diagnosis of current AN, and 27 healthy women without any current or lifetime Axis I diagnosis. Individuals with AN were recruited from the inpatient unit and individuals with BN from the outpatient unit at the Center for Eating Disorders, Department of Liaison Psychiatry and Psychosomatic Medicine, University Hospital of Zurich, Switzerland. Healthy control participants were recruited from local universities, colleges and vocational schools using flyers and electronic advertisements. Participants were female and at least 18 years of age (*M* = 22.6, range 21.4–24) and without past or current neurological disorders or professional knowledge about nutrition (e.g., cook or dietician). Cognitive impairment was ruled out using the Trail Making Test during the study. Data from HC and individuals with AN were previously compared in Milos et al. [2]. For more information on exclusion/inclusion criteria of AN and HC participants, refer to Milos et al. [2].

**Table 1:** Sociodemographic, clinical characteristics and neuropsychological test results for BN, AN and HC

	BN	HC	BN × HC comparison			AN	BN × AN comparison		
	Mean (SD)	Mean (SD)	*t*^a^	*df*^a^	*p* value^a^	Mean (SD)	*t*^a^	*df*^a^	*p* value^a^
*N*, No.	27	27	–	–	–	28	–	–	–
Age, years	24.0 (5.6)	21.4 (2.7)	2.12	37.8	0.040	22.5 (4.2)	−1.1	48.4	0.2777
BMI, kg/m^2^									
Current	20.6 (1.7)	21.5 (2.7)	−1.35	44.4	0.183	15.6 (2.1)	−9.2	46.5	< 0.001
Lowest	17.6 (2.1)	20.4 (2.3)	−4.37	47.8	< 0.001	13.1 (1.8)	−7.7	42.8	< 0.001
Highest	24.3 (2.9)	22.4 (3.3)	2.1	47.9	0.041	20.9 (2.9)	−3.9	44.0	< 0.001
Hunger^b^	28 (30.3)	35.4 (28.4)	−0.86	41.7	0.390	15.6 (19.8)	−1.6	33.0	0.115
Eating disorder inventory									
Total score	159 (98.3)	64.9 (27.2)	4.07	33.8	<0.001	187 (33.8)	1.2	21.2	0.251
Drive for thinness	22 (11.8)	6.07 (4.7)	5.57	22.0	< 0.001	26.3 (7,7)	1.4	28.8	0.175
Bulimia	23.5 (11.4)	14.5 (9.2)	2.8	33.5	0.007	32.7 (6.6)	3.2	26.8	0.003
Body dissatisfaction	22.4 (17.7)	8.07 (5.3)	3.4	20.2	0.003	31.4 (9.6)	2.0	25.6	0.054
Ineffectiveness	22.6 (17.9)	9.0 (4.6)	3.2	19.6	0.004	18.4 (4.7)	−1	19.8	0.325
Perfectionism	16.4 (9.0)	7.2 (5.1)	4.0	26.3	< 0.001	29.5 (7.7)	5.1	35.2	< 0.001
Interpersonal distrust	14.1 (11.7)	9.5 (3.9)	1.6	20.8	0.113	23.0 (7.4)	2.9	28.4	0.006
Interoceptive awareness	21.9 (14.2)	3.0 (2.7)	5.7	18.9	< 0.001	9.1 (7.6)	−3.6	25.4	0.001
Maturity fears	16.3 (14.4)	7.6 (3.7)	2.6	19.7	0.018	16.5 (6.3)	0.04	23.0	0.967
Beck depression inventory II	21.6 (12.0)	3.0 (3.2)	6.6	19.8	< 0.001	24.6 (10.3)	0.9	35.6	0.385
State-trait anxiety inventory									
Trait	55.6 (8.7)	32.7 (7.6)	9.4	37.7	< 0.001	58.2 (8.02)	1.05	39.1	0.298
Figure rating scale^c^									
Current figure	25.7 (10.2)	22.7 (8.1)	1.2	49.3	0.230	14.2 (9.8)	−4.1	48.7	< 0.001
Ideal figure	12.3 (6.7)	19.3 (4.9)	−4.3	47.4	< 0.001	11.0 (6.7)	−0.7	48.3	0.496
Trail making test^d^									
Errors A	0.6 (0.9)	0.07 (0.3)	2.9	30.6	0.007	0.17 (0.4)	−2.3	36.1	0.029
Errors B	1.1 (2.1)	0.07 (0.3)	2.6	26.8	0.015	0.08 (0.3)	2.6	27.0	0.015

*BN* bulimia nervosa, *HC* healthy controls, *AN* anorexia nervosa, *BMI* body mass index

^a^Group comparisons were done using *t*-test

^b^Measured using a one-item visual scale (Farooqi et al. [19])

^c^Pruis and Janowsky [17], Stunkard et al. [18]

^d^Reitan [21]; Reitan [22]

*Standardized diagnostic interview*. The presence of past and current psychiatric disorders, including ED, was determined with the Structured Clinical Interview for DSM-IV Axis I Disorders (SCID-I)^1^ [10].

*Standardized clinical* questionnaires. The severity of depressive symptoms was assessed using the Beck Depression Inventory (BDI) [11–13] and eating disorder symptoms and features were assessed using the Eating Disorder Inventory (EDI) [14]. Besides yielding a total score, EDI also allows for the assessment of ED related symptoms such as drive for thinness, bulimia, body dissatisfaction, ineffectiveness, perfectionism, interpersonal distrust, interoceptive awareness, and maturity fears. Trait anxiety was measured by the State-Trait Anxiety Inventory (STAI) [15, 16].

*Figure rating scale*. First, subjects watched a video of a functionalized female body. When they clicked on “play”, this body became thicker, on a scale from 01 to 80. They could pause and rewind the video at any time. First, they indicated how they saw their body at the moment and then how they would like to be [17, 18].

*Hunger assessment*. A one-item hunger scale [19] was administered to assess the current hunger state. When presented with a visual analogue scale ranging from not full/not hungry, to extremely full/satiated, participants were asked to indicate their hunger level [20].

*Trail making test*. Cognitive performance was examined using the paper-and-pencil version of the Trail Making Test (TMT) for attention and set-shifting [21, 22]. This Test consists of two parts, each preceded by a short exercise. In the first part, participants were asked to draw lines connecting the numbers from 1 to 25 as quickly as possible and without lifting the pencil from the paper. The beginning (1) and the end (25) are indicated. At this time, the timer is stopped, and the number of mistakes is counted. If a mistake (omitting a number or lifting a pencil) is found, the respondent is made aware of this and must continue from the last correctly marked number, while the timer continues to run. Time to complete the task and the number of errors were recorded. The second part had the same procedure with the difference that the numbers from 1 to 13 and the letters from A to L were on the sheet. Participants were then told to connect numbers with letters with numbers with letters and so on whereby both numbers and letters had to be in the correct order. That is, a line should be drawn from 1 to A, from A to 2, from 2 to B, from B to 3, and so on up to 13. Again, total time to complete the task and number of errors were recorded.

*Portion estimation task*. As in Milos et al. [2] participants performed a computer-based task that required the size of sequentially displayed meal portions to be estimated on a visual analogue scale (0 = small, 100 = large). The meal pictures consisted of a breakfast meal (bread, butter, jam, yoghurt or muesli, orange juice), a main course A (meat, risotto, broccoli, fruit salad, with or without ice cream) and a main course B (lasagna, salad, applesauce with vanilla cream). In main course A, macro nutriments of the meal were better recognizable than in main course B. Each meal was depicted by six different serving sizes: 1/8, ¼, ½, 1, 1 ½, 1 ¾ (pictures of the apportioned meals are available on Milos et al. [2]). A single presentation of each of the 18 meal-portion pictures was incorporated into two blocked conditions (general, intent-to-eat). In the general condition, participants were simply instructed to estimate the size of the meal portion in general; in the intent-to-eat condition, participants were instructed to estimate the size of the meal portion while imagining they were supposed to eat the pictured meal. The sequences of pictures and conditions were randomly determined. After participants finished estimating the meal portion sizes, the full set of 18 meal pictures was displayed on the screen in a randomized arrangement (6 pictures from course A, then 6 pictures of course B, and then 6 pictures of breakfast) and participants were asked to order the depicted meals according to their size, beginning with the smallest one. Meal portion sizes were determined by the local nutritional advisor from the University Hospital of Zurich (Division of Endocrinology, Diabetes and Clinical Nutrition) according to guidelines of the Swiss Society for Nutrition. Carbohydrates, proteins and fat were ideally balanced in each meal. Based on this information and the assumption that 600–700 kcal represents a normal meal for 18–30 years old women [23], a normal meal portion (size 1) was defined and used as the basis for deriving the other meal portion sizes.

*Procedure*. Participants were instructed to eat a meal 2 h prior to the experiment, and refrain from eating after that, in order to minimize the effect of hunger on meal portion size estimates [24]. Participants underwent the diagnostic interview, followed by the neuropsychological test, standardized clinical questionnaires and the experimental task, in that sequence. The entire procedure took approximately 3 h.

*Data analysis*. T-tests comparing the clinical and cognitive assessments and exploratory correlation analyses were performed using R [25] (Version 1.3.1073). Mixed models of the portion estimation task were calculated using IBM SPSS Statistics 22 (IBM Corp., Armonk, NY, USA). To avoid retesting of previously reported contrasts, two separate models were calculated to compare the individuals with BN to healthy controls (model I) and to individuals with AN (model II). These two models included Group (bulimia nervosa (BN) vs. healthy controls (HC) for model I, bulimia nervosa (BN) vs. anorexia nervosa (AN) for model II), Intent (general condition, intent-to-eat condition), Portion size (1/8, 1/4, 1/2, 1, 1 1/2, 1 3/4) as fixed factors. Subject was treated as a random intercept with a variance component covariance structure. The trial sequence was modelled as repeated measurements with a diagonal covariance structure. Bonferroni–Holm corrected pairwise comparisons of the estimated marginal means were used as post-hoc tests when applicable.

*Exploratory analyses*. We further explored the relationship of portion estimation and eating disorder symptoms across all participants. We created a single portion estimation item (a means of all estimation trials) and explored Pearson’s correlation analyses between this item and each subscale of the EDI, hunger assessment, STAI and BDI.

### Results

*Comorbidity in the BN group according to SCID Questionnaire.* Previous (not current) AN (*n* = 5, 35.7%), current major depression (*n* = 7, 50.0%), previous major depression (*n* = 8, 57.1%), previous dysthymia (*n* = 1, 7.1%), current depressive disorder not specified (*n* = 2, 14.3%), previous depressive disorder unspecified (*n* = 2, 14.3%), previous alcohol dependence/abuse (*n* = 2, 14.3%), previous obsessive–compulsive disorder (*n* = 1, 7.1%), and current post-traumatic stress disorder (*n* = 1, 7.1%).

*Demographics and standardized clinical questionnaires.* In the BN vs HC comparison, no significant difference was found for current BMI (*p* = 0.1834). BN had significantly lower lowest (*p* < 0.001), and higher highest (*p* = 0.041) BMI compared to HC. In comparison with AN, BN had significantly higher current (*p* < 0.001), lowest (*p* < 0.001), and highest (*p* < 0.001) BMI (Table 1).

With relation to clinical measures, BN had significantly higher BDI scores in comparison with HC (*p* < 0.001), while no significant difference was found between BN and AN (*p* = 0.385). Similar results were found for trait STAI, with BN showing significantly higher scores than HC (*p* < 0.001) but not different ones from AN (*p* = 0.298). Except for interpersonal distrust (*p* = 0.113), BN showed significantly higher scores in comparison with HC (all *p* values < 0.05, see Table 1 for more details) in EDI total score and EDI subscales. Compared to AN, BN showed significantly lower scores for the bulimia, perfectionism, and interpersonal distrust EDI subscales (all *p* values < 0.01) and higher interoceptive awareness (*p* < 0.001). No significant difference was found for EDI total score (*p* = 0.251), drive for thinness (*p* = 0.175), ineffectiveness (*p* = 0.325), and maturity fears (*p* = 0.967) (Table 1).

*Figure rating scale.* While no difference was found between BN and HC for current figure (*p* = 0.230), and between BN and AN for ideal figure (*p* = 0.496), BN’s ideal figure scores were lower than HC (*p* < 0.001) and BN’s current figure scores were higher than AN (*p* < 0.001) (Table 1).

*Hunger assessment.* No significant difference was found in any model for hunger assessment (Model I, *p* = 0.390; Model II, *p* = 0.115) (Table 1).

*Neuropsychological tests*. All subjects were able to accurately order the meal pictures according to their portion size when all pictures was visible and a direct comparison was possible. Individuals with BN showed significant worse food unrelated cognitive performance at TMT than HC and individuals with AN (Table 1).

**Fig. 1:**
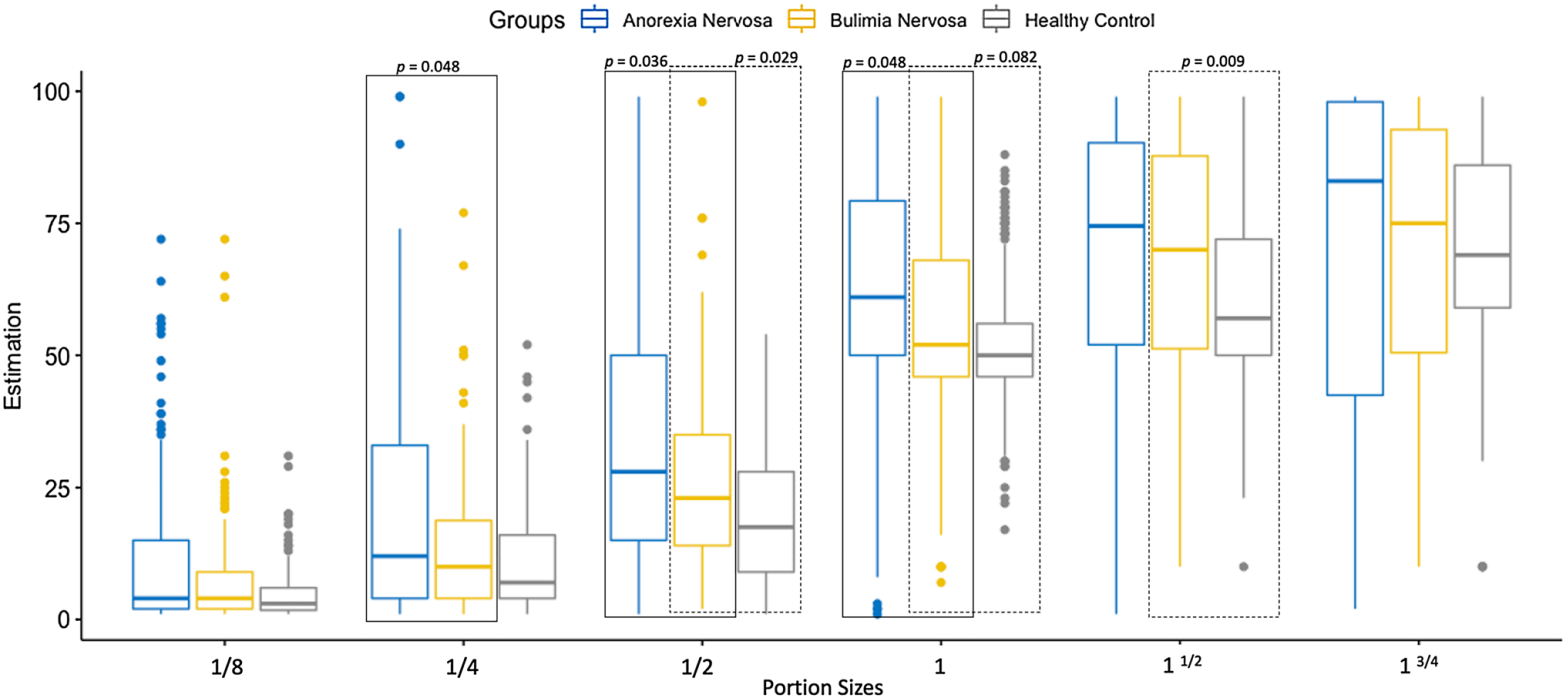
Bar graphs of modeled mean estimation scores including standard errors across 3 Meal types (breakfast, main course A, main course B) by Portion size (1/8, 1/4, 1/2, 1, 1 1/2, 1 3/4) and Group (AN, BN, HC) across Intent (general condition, intent-to-eat condition)

*Estimation of meal portion sizes—model I—BN vs HC*. For the comparison between BN and HC, a significant difference was found for the interactions group × portion size (F_(5, 1820)_ = 4.477, *p* < 0.001) and group × meal (F_(2, 1820)_ = 14.75, *p* < 0.001). A significant difference was also found for intent (F_(1, 1820)_ = 18.32, *p* < 0.001). Post hoc analyses revealed that for the portion sizes ½, and 1 ½, individuals with BN had higher ratings compared to HC (*p* values < 0.05). For portion size of 1, a trend was observed (*p* = 0.082), with BN also having higher rates than HC. See Fig. 1 for details. While no differences were found between groups for intent (*p* = 0.639), participants (BN and HC included) rated portions larger in the intent-to-eat compared to the general condition (*p* < 0.001) (Fig. 1).

*Estimation of meal portion sizes—Model II—BN vs AN.* For the comparison between BN and AN, a significant difference was found for the interactions Group x Portion Size (F_(5, 1855)_ = 2.682, *p* = 0.020), and intent × portion size (F_(5, 1855)_ = 3.009, *p* = 0.010). Post hoc analyses revealed that for the portion sizes ¼, ½ and 1, individuals with BN had lower ratings compared to AN (*p* values < 0.05). See Fig. 1 for details. Participants with BN and AN also rated portions 1/8, ¼ and ½ larger in the intent-to-eat compared to the general condition (*p* values < 0.050). Differences for the regular size portion (i.e., 1) approached significance (*p* = 0.051). No differences were found between groups for intent (Fig. 1).

*Exploratory correlation analyses*. To examine the relationship between portion estimation and eating disorders symptoms, exploratory Pearson’s correlation analyses were calculated for BN and HC (see Supplemental Material for further details). Higher mean portion estimation significantly correlated with higher EDI total score (*r* = 0.27, *p* = 0.02) and particularly with the EDI subscales drive for thinness (*r* = 0.42, *p* < 0.001), bulimia (*r* = 0.29, *p* = 0.015), and perfectionism (*r* = 0.36, *p* < 0.01). Further, mean portion estimation was associated with higher BDI (*r* = 0.38, *p* < 0.01) and STAI scores (*r* = 0.29, *p* = 0.012) and lower self-reported hunger (*r* = −0.49, *p* < 0.001). There was no evidence for a relationship between mean portion estimation and BMI (*p* > 0.20) (Fig. 1). In multiple regressions for the total portion estimation score for the various significant findings (see Supplemental Materials), drive for thinness was the most reliable predictor of portion estimation (*p* = 0.002).

### Discussion

Estimating food portion sizes is a challenge for individuals with EDs [2]. Evaluating food portion estimation in individuals with bulimia nervosa is important because it can provide further insight into the maintenance of the disorder and guide treatment options. The present study investigated meal portion size estimation in individuals with BN compared to (1) healthy controls and (2) individuals with AN using computerized stimuli. In partial agreement with our hypotheses, the ratings of individuals with BN were significantly larger for some portion sizes, compared to healthy controls and significantly smaller compared to individuals with anorexia nervosa, regardless of intent (i.e., independently of who the meal was meant for).

Individuals with BN rated intermediary portion sizes (i.e., portion sizes ½, 1 and 1 ½) larger than healthy controls who had no problems with the realistic estimation of intermediary (normal) portion sizes. For individuals without eating disorders it seems to be easier to identify smaller and bigger portions, likely because they are a variation of the normal ones. Our results show that there are no significant differences between individuals with BN (normal-weight condition) and with AN (underweight condition) for extreme portion sizes of the task. In the past, we hypothesized that underweight could explain portion sizes overestimation. Our current results show that, although underweight may play an important role (individuals with AN still rated portions smaller than individuals with BN), there are other factors driving the differences between the two ED types. The presence of an eating disorder could explain why individuals with BN (and AN) have difficulties in estimating portion sizes. Our results suggest that eating disorder symptoms (EDI total score) and, more specifically, the EDI subscale “drive for thinness”, and not BMI, were most strongly associated with higher portion estimation. This was also associated with lower self-reported hunger.

For the portion sizes ¼, ½ and 1, individuals with BN had lower ratings compared to AN. This underlines that in AN food ingestion is linked to a specific psychopathology probably related to the underweight condition. Perfectionism, Interpersonal Distrust, Interoceptive Awareness (EDI subscales) and the current figure item scores were lower for BN individuals compared to AN. Tabri et al. [26] found that shape/weight concerns (key aspect of the transdiagnostic cognitive-behavioral model of eating disorders [5] had a reciprocal relationship with restrictive eating among women diagnosed with AN or BN. Individuals with BN frequently have very restrictive eating during the day in order to lose weight [26], such that they have an increased hunger feeling in the evening, potentially leading to binge [4]. This restrictive trait of individuals with BN (also a characteristic for individuals with AN) could explain their partial meal size overestimation in our study. This could also be related to a specific weight-gain anxiety, which is usually less pronounced in BN that in AN. Further investigation is needed.

Participants with BN and AN also both rated portions 1/8, ¼ and ½ larger in the intent-to-eat compared to the general condition, what could also be a consequence of the transdiagnostic model of EDs [5], with BN and AN sharing some psychopathological and maintenance factors. The overestimation of food portion sizes in BN seems to reflect one aspect of the general cognitive distortions typically seen also in AN [27, 28], where perception is not only disturbed in relation to interoceptive awereness [29, 30], but also in relation to non-bodily external objects such as food portion size. The misperception of the own body and disorder-related objects in BN and AN might therefore share a common pathway in their pathogenesis. Stronger effects in AN vs. BN can be interpreted as a distortion effect related to the condition immediately before the ingestion of food and the fear of gaining weight typical for AN, and further research is required to confirm this hypothesis.

BN and AN/HC subjects in our study differed in their food-unrelated cognitive performance on TMT, confirming the findings in Vall et al. [31], who concluded that higher personal perfectionism as in AN subjects seem to predict superior performance, showing similar performances as HC. The impulsivity and inattention in individuals with BN [32, 33] could be related to worse food-unrelated cognitive performances.

Knowing that individuals with active ED (or going through a relapse) perceive smaller food portions as appropriate or ‘normal’ [34], according to the findings of our study a therapeutic approach could be the frequent visual exposure to increasing portions of food (monitoring the changes in portion size estimation throughout the course of the treatment), in order to recalibrate visual perceptions of what a ‘normal’ sized portion of food looks like [3, 34, 35].

The field could benefit from future longitudinal studies investigating the relationship between our findings and the habitual better prognosis of BN compared to AN [36]. Furthermore, correlations between meal portion overestimation and impulsivity/inattention in individuals with BN could also be considered in the development of specific interventions targeting eating behavior in BN and should be investigated. The aspect of weight-gain anxiety in AN and BN should also be taken in account in future studies, with a specific extension of the present task.

In conclusion, the estimation of food portion sizes in BN seems to be altered compared to HC, but to a lesser extent than in AN. The presence of an eating disorder could help explain why individuals with BN (and AN) have difficulties in estimating portion sizes. A therapeutic option could include the frequent visual exposure to increasing portions of food, what may serve to recalibrate visual perceptions of what a “normal-sized” portion of food looks like.

### Strength and limits

Although very important for understanding the etiology of BN, no study has evaluated food portion estimation in this disorder so far and so we are the first ones in this field investigating meal portion size estimation in individuals with BN compared to healthy controls and individuals with AN, allowing a comparison within these groups. This study has also some limitations. First, although we used mixed-model analyses, which allows for larger degrees of freedom, the sample size of the present study was small. Second, though custom-created meal is an appropriate methodology that allows to avoid cultural bias [37, 38] when comparing patients across cultures, the food stimuli used cannot be considered as standardized and may not be generalizable. Furthermore, the stimuli are computerized pictures presented on a computer screen, it is here important to consider, that this is a clear abstraction away from real food conditions. Further studies should explore portion size estimation using actual food in both a laboratory and real setting. Third, since correlation and multiple regression analyses were exploratory, with no correction for multiple comparisons, these results should be interpreted with caution. Finally, it is important to note that these results were partially for inpatients who sought/agreed to specialized support for their severe condition. Future studies should investigate whether findings are replicated with outpatients.

### What is already known on this subject?

A previous study indicated that individuals with AN tend to overestimate food portion sizes compared to HC and whether this estimation is modulated by an intent-to-eat instruction (where patients are asked to imagine having to eat the presented meal). Subjects with AN estimated the size of small and medium meal portions (but not large meal servings) as being significantly larger, compared to estimates of HC. The overestimation of small meal portions by AN subjects was significantly greater in the intent-to-eat, compared to the general condition (in which they were instructed to rate portions as if someone else was supposed to eat them).

### What this study adds?

No study has evaluated food portion estimation in BN as far. It is important to assess portion estimation in BN to find out if individuals with BN share food-related cognitive anomalies with individuals with AN, according to the transdiagnostic theory of ED (Fairburn et al. [5]), since, following weight-recovery treatment, individuals with AN may switch to a diagnosis of BN. The estimation of food portion sizes in BN seems to be altered compared to HC, but to a lesser extent than in AN. The presence of an ED could help explain why individuals with BN (and AN) have difficulties in estimating portion sizes.

## Supplementary Information

Below is the link to the electronic supplementary material.

Supplementary file1 (DOCX 1218 KB)

## Data Availability

The datasets generated and/or analyzed during the current study are available from the corresponding author on reasonable request.

## Abbreviations

AN: Anorexia nervosa
BN: Bulimia nervosa
ED: Eating disorders
HC: Healthy controls
EDI: Eating disorder inventory
BDI: Beck depression inventory
STAI: State-trait anxiety inventory
SCID-I: Structured clinical interview for DSM-IV axis I disorders
TMT: Trail making test

## Footnotes

1 The SCID I was used since data were collected previous to 2013.
