# Supplementary material for: Estimation of meal portions in bulimia nervosa compared to anorexia nervosa and healthy controls
Source: Eat Weight Disord. 2022 May 19;27(7):2665–72. doi: 10.1007/s40519-022-01410-w (PMC9556356; doi:10.1007/s40519-022-01410-w)
Supplement: Supplementary file 1 — Supplementary file1 (DOCX 1218 KB) [file 40519_2022_1410_MOESM1_ESM.docx]

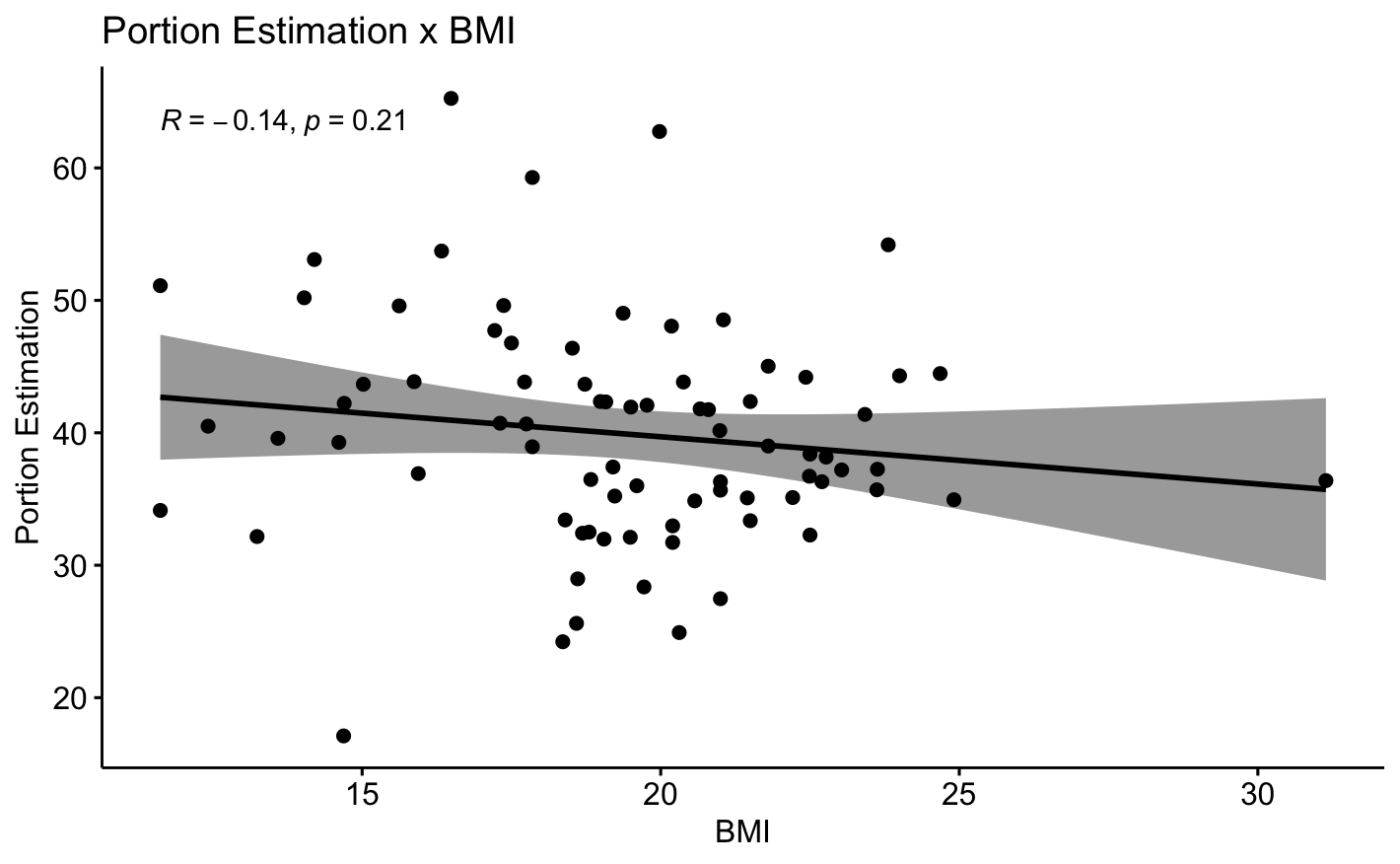

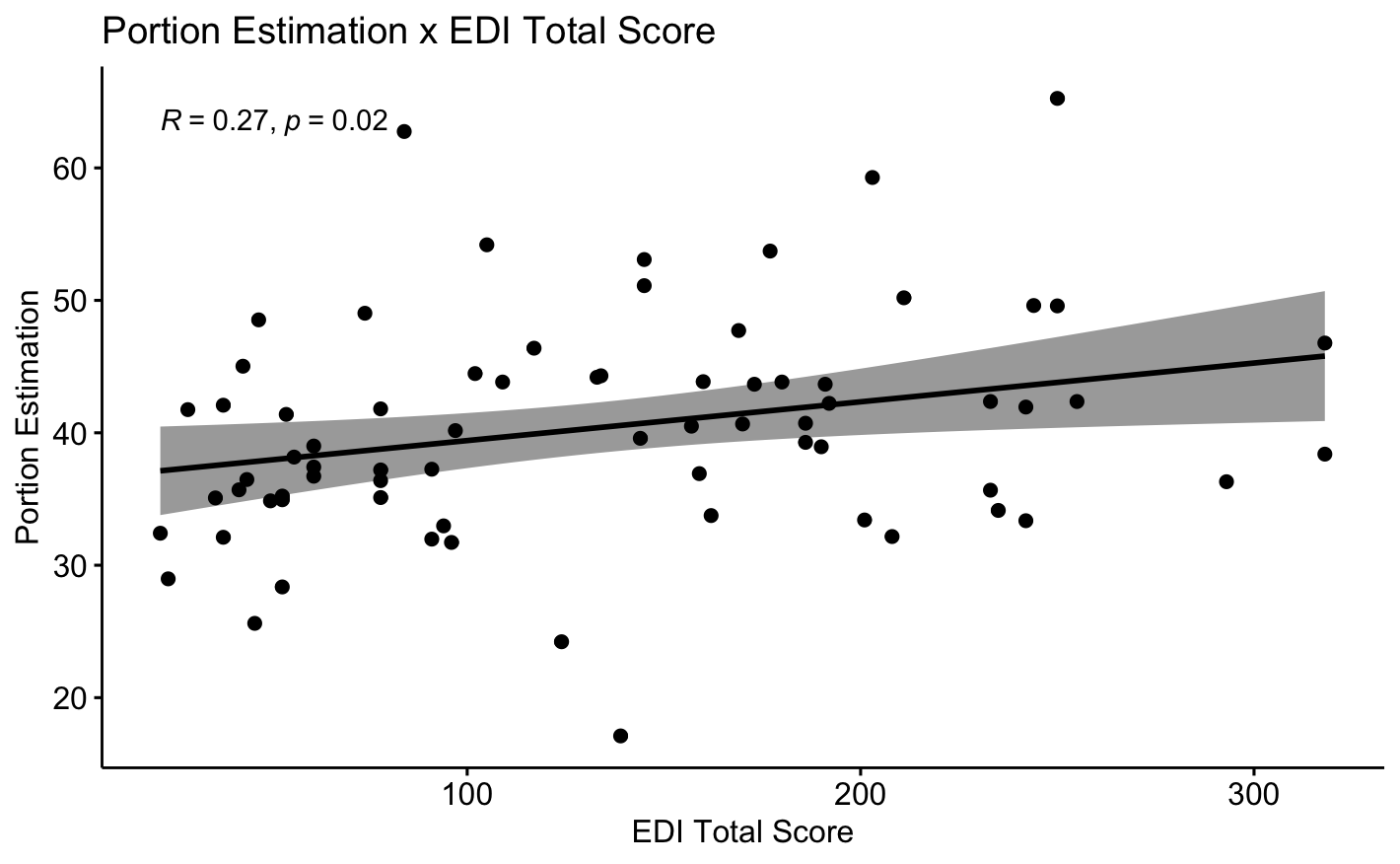

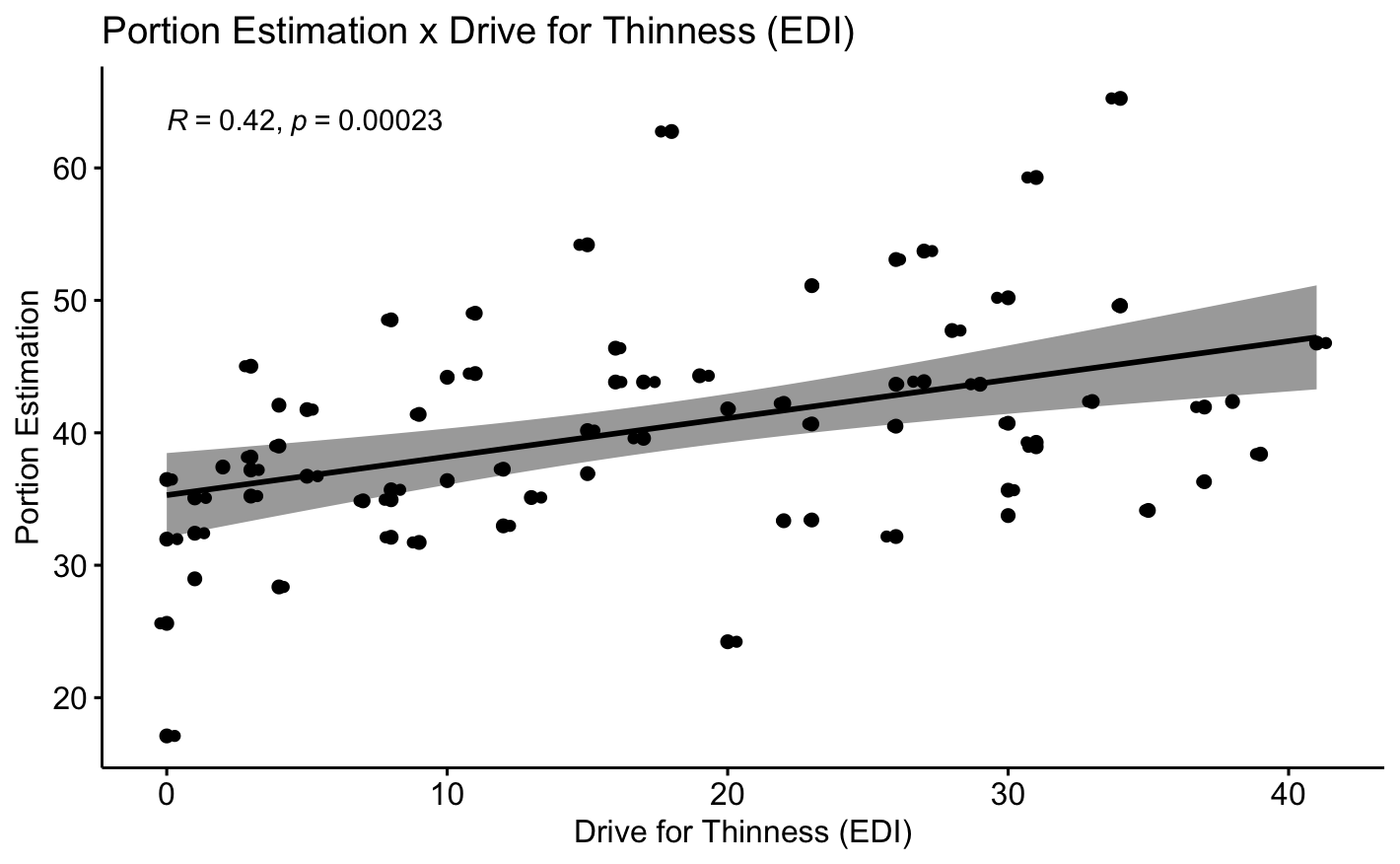

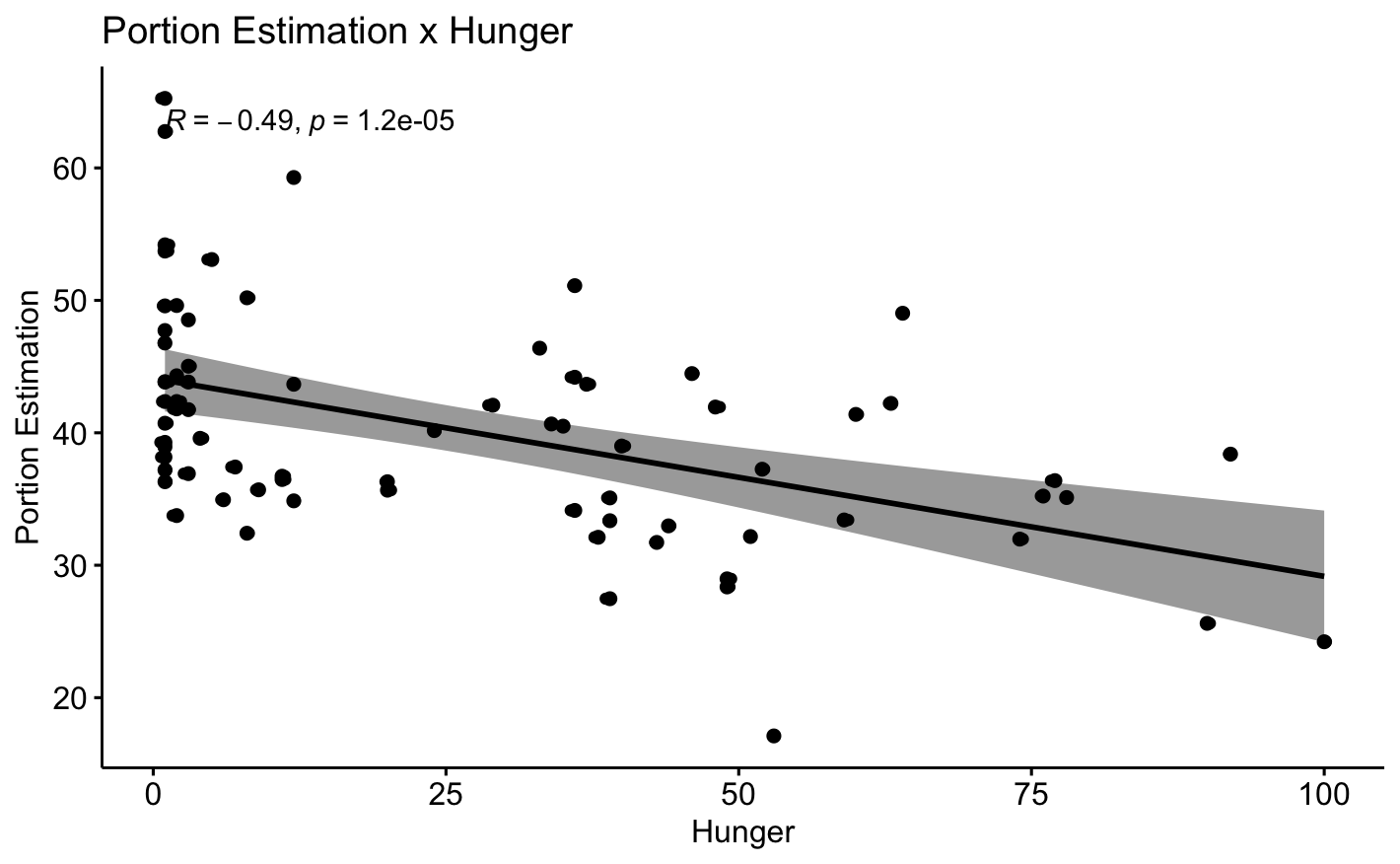

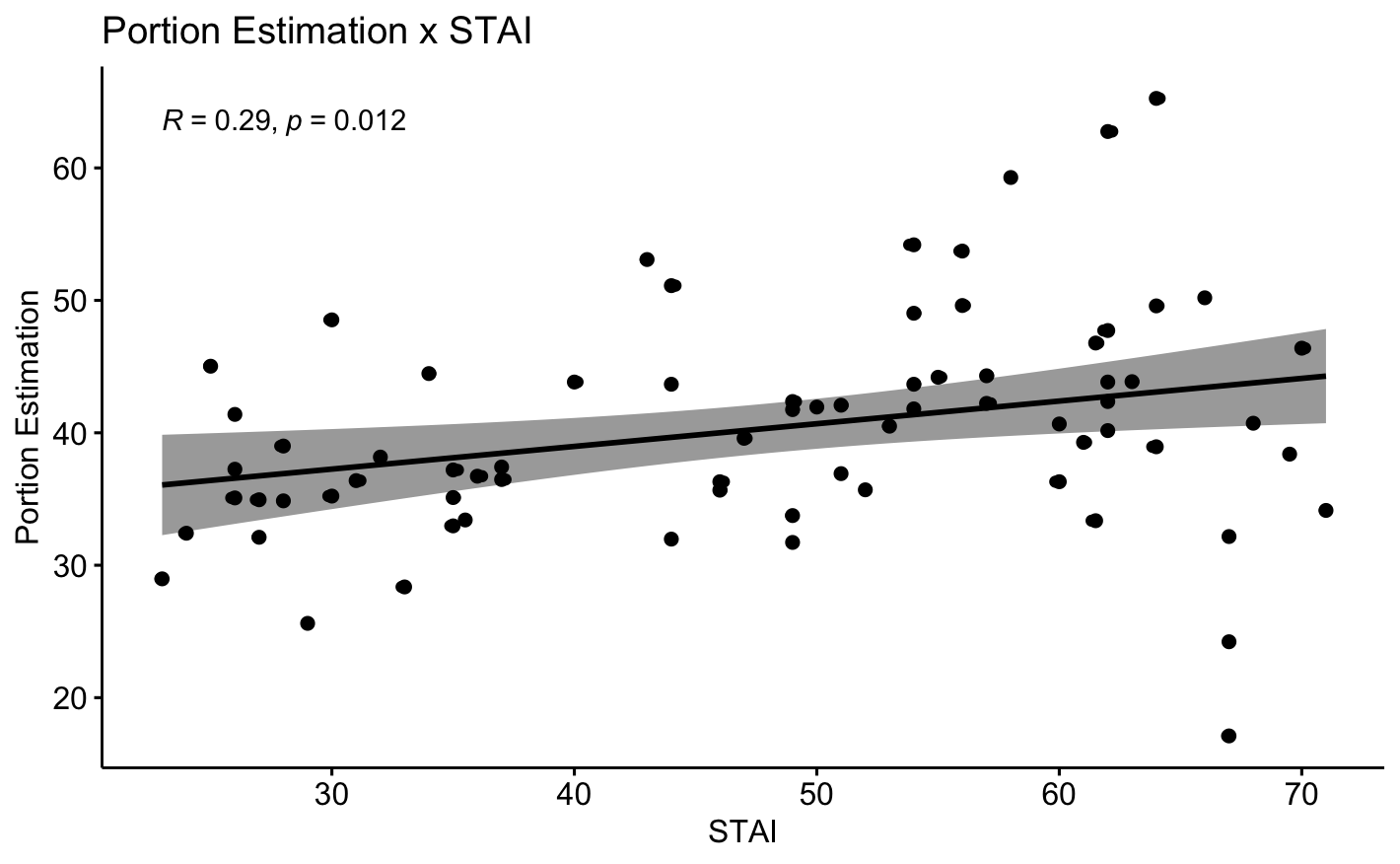

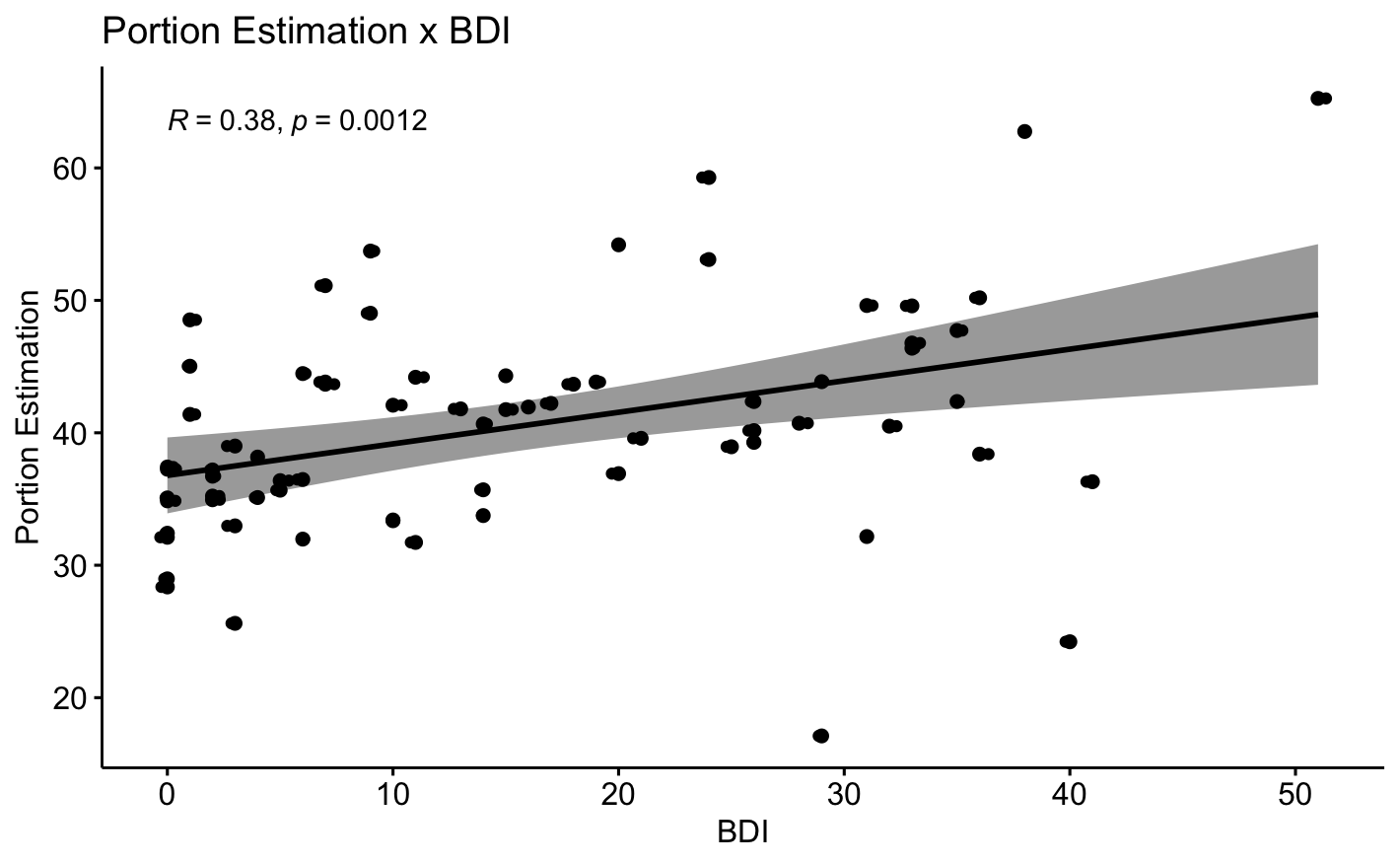

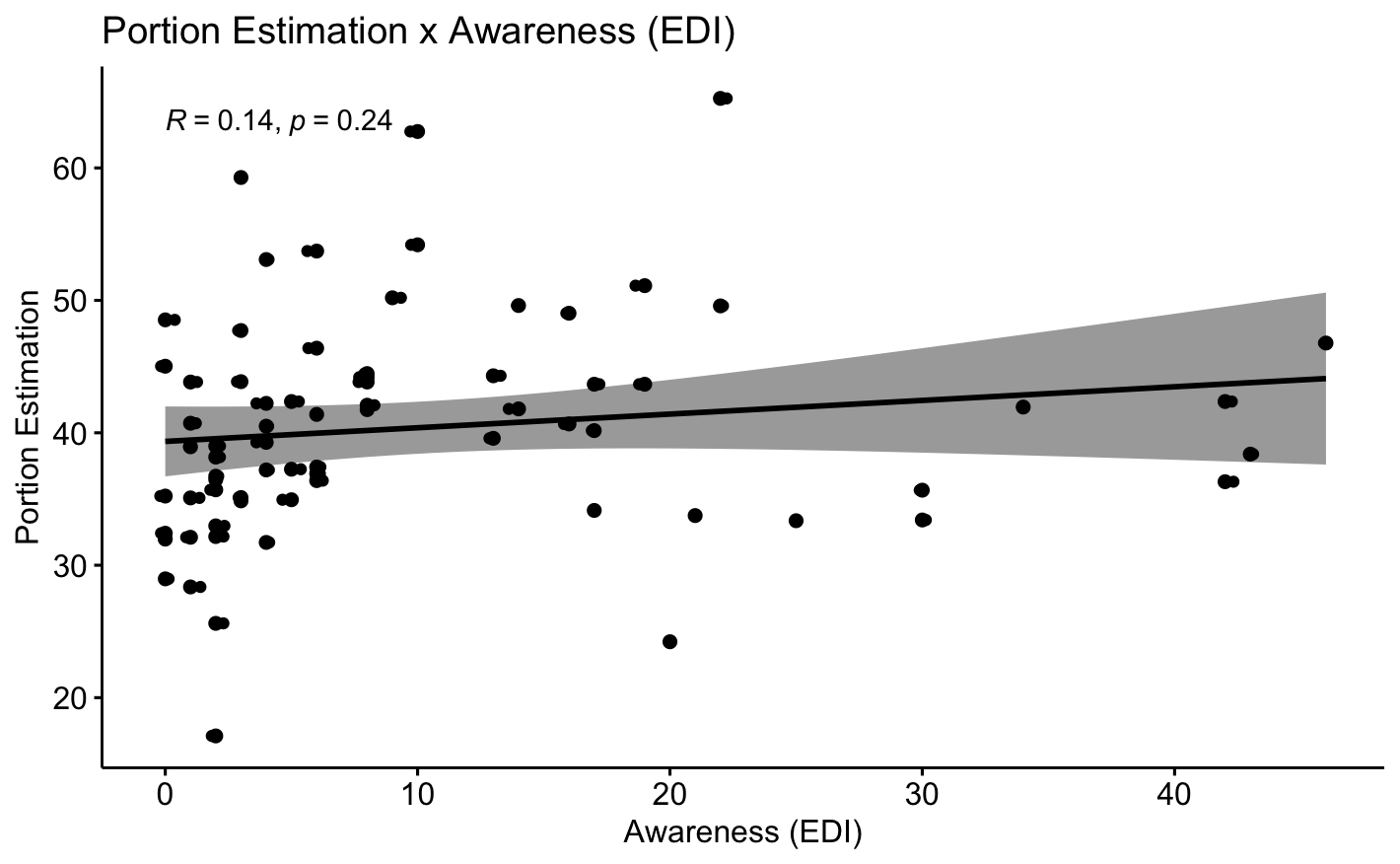

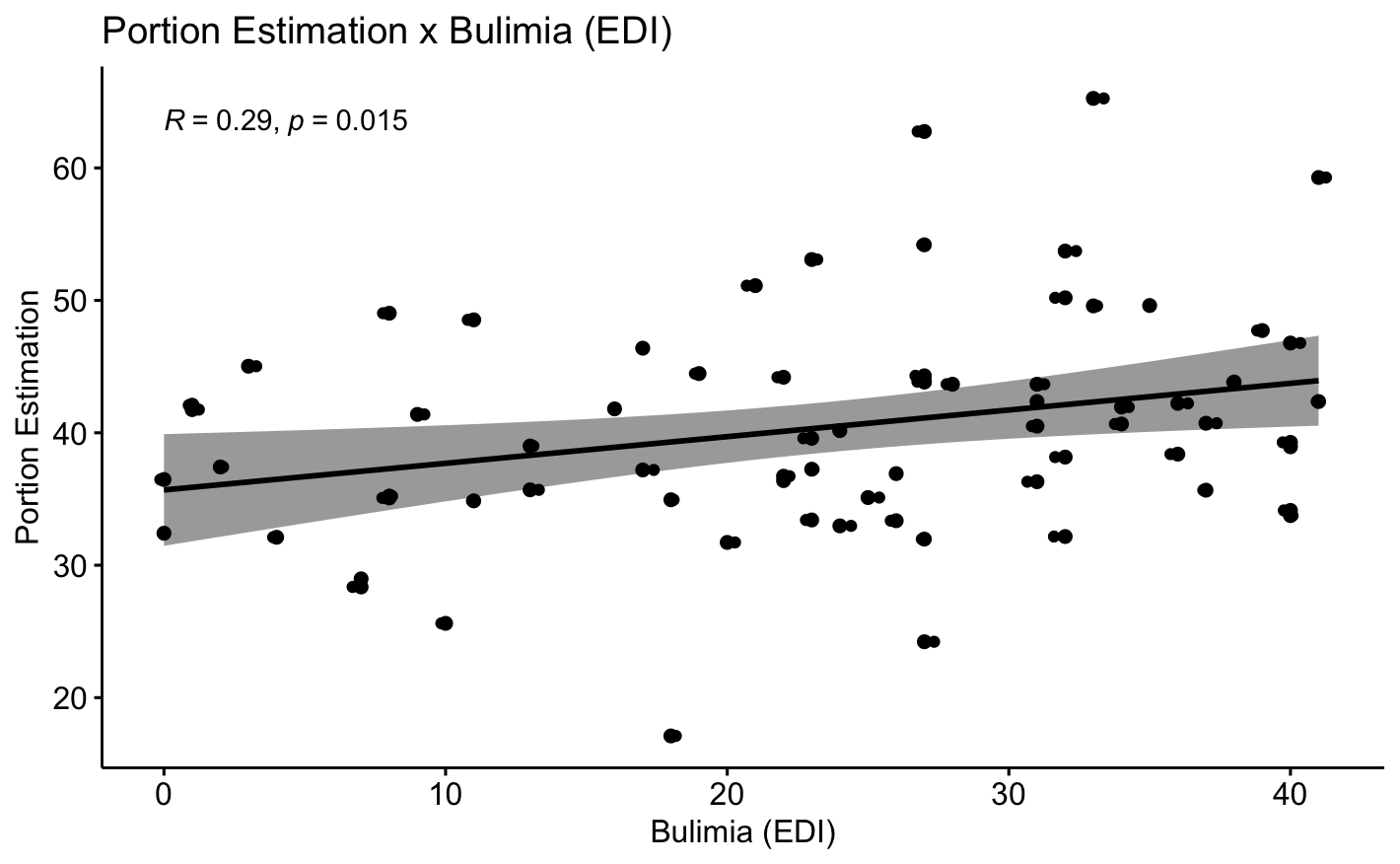

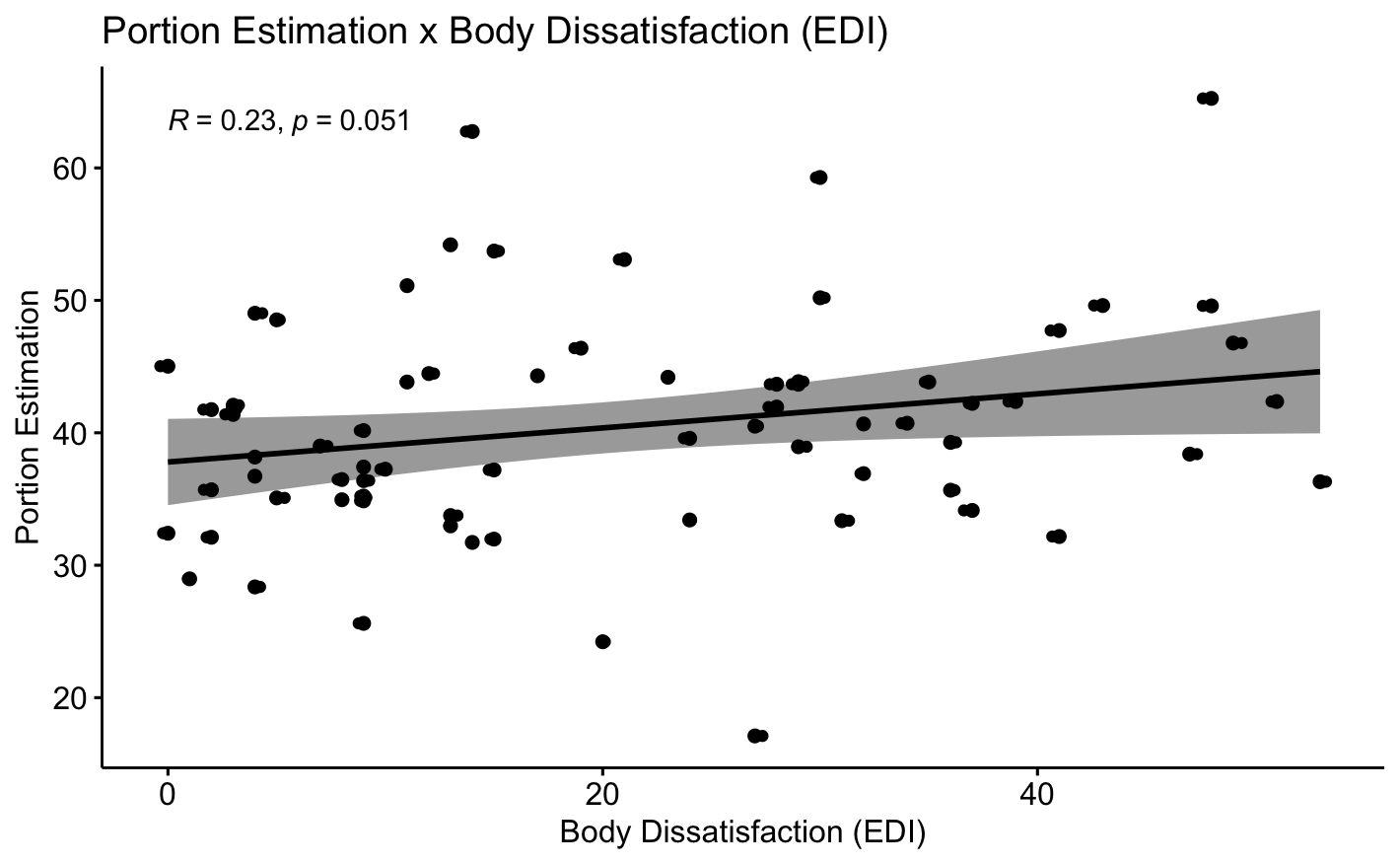

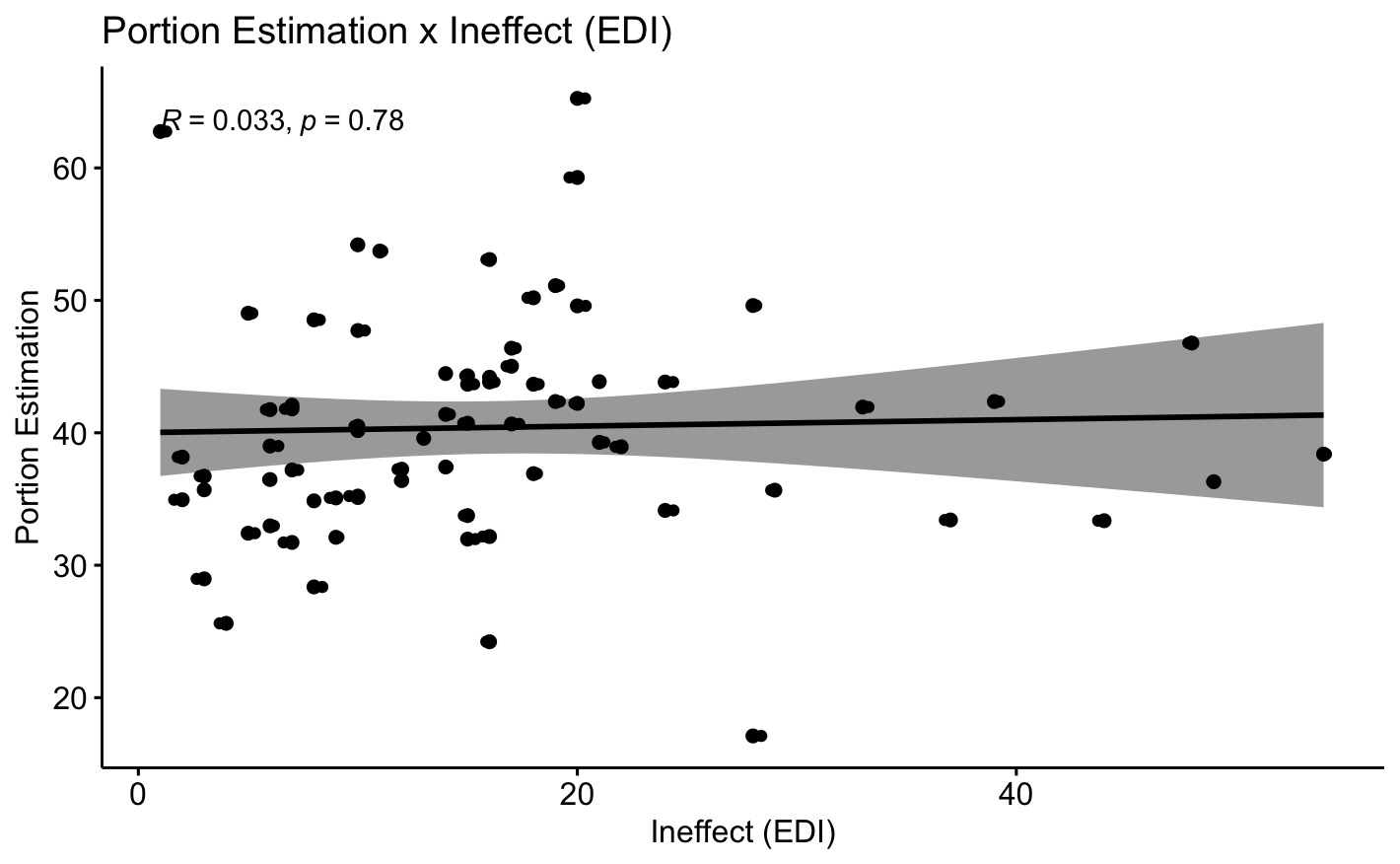

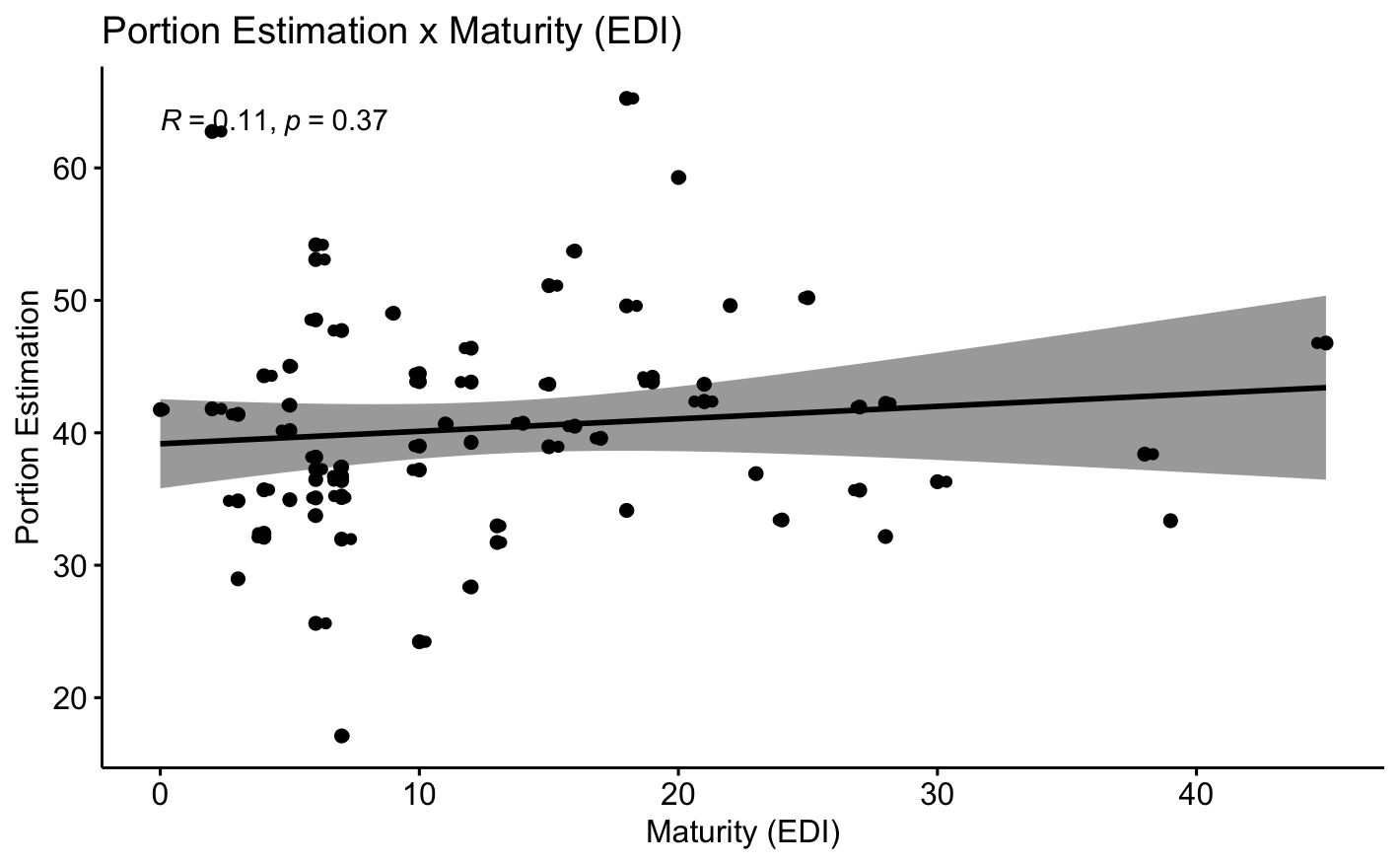

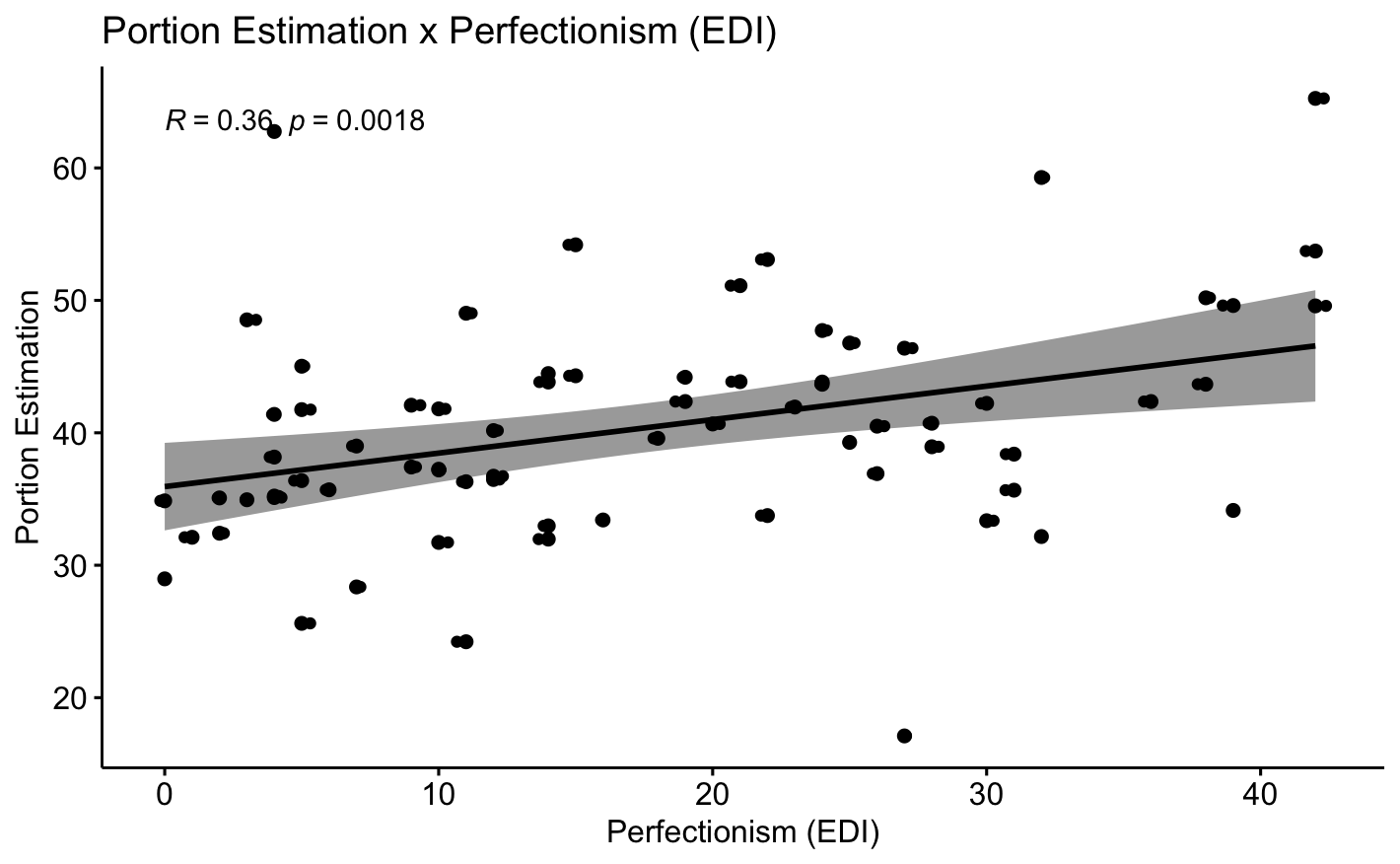

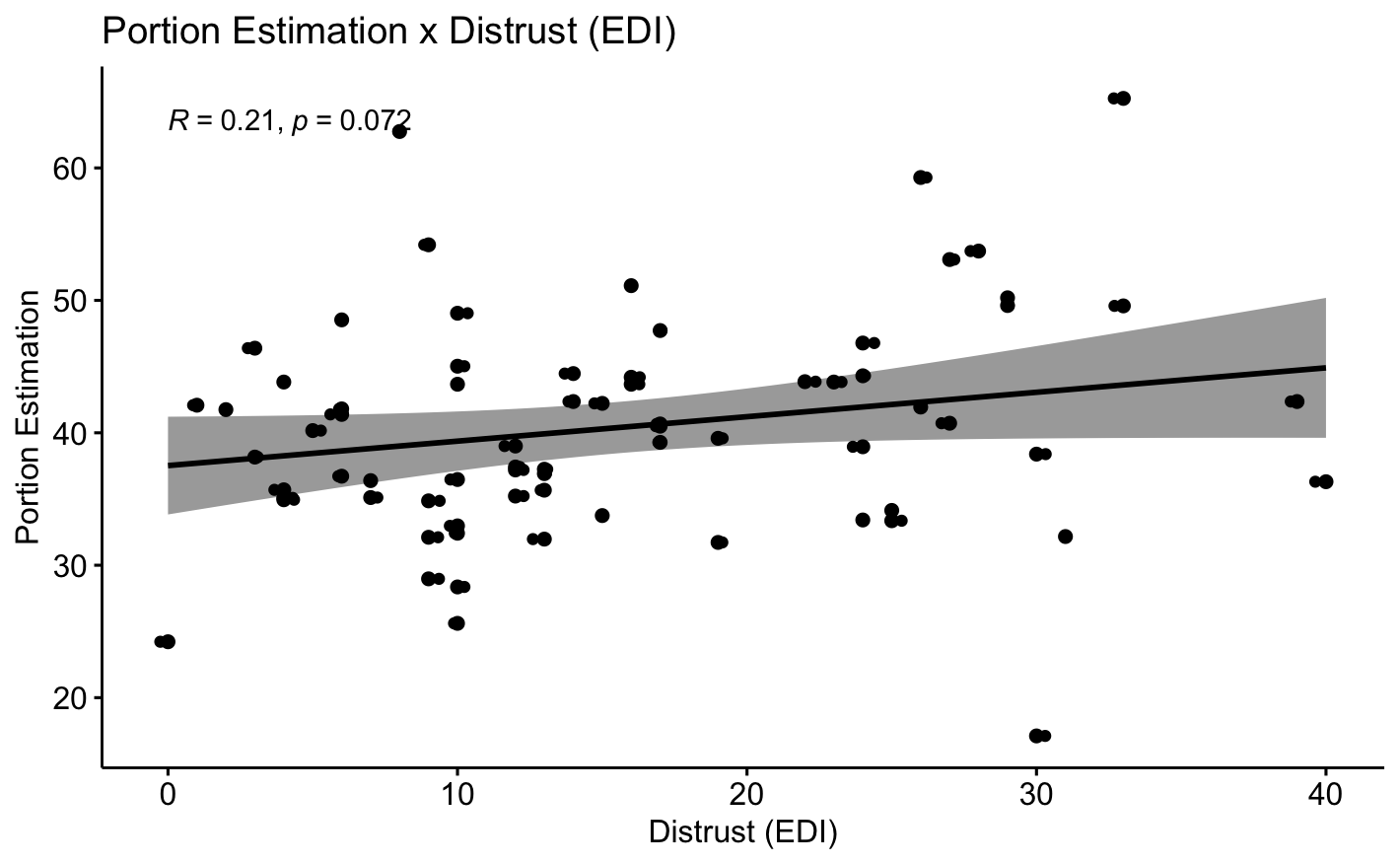


Relationship between portion estimation and EDI, hunger, STAI, BDI

───────────────────────────────────────────

                                  Model 1                Model 2

───────────────────────────────────────────

  (Intercept)                     40.49 ***             37.25 ***

                                        (0.78)                   (2.10)

  EDI_total                      -6.95 **           -8.80 **

                                        (2.37)                   (2.55)

  EDI_drivethin                7.01 **                 7.21 **

                                        (2.19)                   (2.40)

  EDI_bulimia                  -0.95                    -0.11

                                        (1.45)                   (1.59)

  EDI_perfect                    3.61 *                   6.15 **

                                        (1.55)                   (2.07)

  Hunger -2.55 ** -2.75 **

(0.85) (0.85)

STAI -1.26 -2.10

(1.71) (2.20)

BDI 1.86 2.66

(1.64) (1.69)

  Group BN                                             5.30

                                                                 (2.95)

  Group HC                                               4.79

                                                                  (3.84)

────────────────────────────────────────────

  N                                     71                       71

  R2                                   0.45                 0.48

────────────────────────────────────────────

 All continuous predictors are mean-centered and scaled by 1standard deviation.

 *** p < 0.001; ** p < 0.01; * p < 0.05.
